# An Unconventional View of T Cell Reconstitution After Allogeneic Hematopoietic Cell Transplantation 

**DOI:** 10.3389/fonc.2020.608923

**Published:** 2021-02-18

**Authors:** Hana Andrlová, Marcel R. M. van den Brink, Kate A. Markey

**Affiliations:** ^1^ Department of Immunology, Sloan Kettering Institute, Memorial Sloan Kettering Cancer Center, New York, NY, United States; ^2^ Adult Bone Marrow Transplantation Service, Department of Medicine, Memorial Sloan Kettering Cancer Center, New York, NY, United States; ^3^ Division of Medicine, Weill Cornell Medical College, New York, NY, United States

**Keywords:** immune reconstitution, unconventional T cells, microbiome, allogeneic transplantation, mucosal invariant T cells (MAIT) cells, γδ T cells, invariant NK T (iNKT) cells

## Abstract

Allogeneic hematopoietic cell transplantation (allo-HCT) is performed as curative-intent therapy for hematologic malignancies and non-malignant hematologic, immunological and metabolic disorders, however, its broader implementation is limited by high rates of transplantation-related complications and a 2-year mortality that approaches 50%. Robust reconstitution of a functioning innate and adaptive immune system is a critical contributor to good long-term patient outcomes, primarily to prevent and overcome post-transplantation infectious complications and ensure adequate graft-versus-leukemia effects. There is increasing evidence that unconventional T cells may have an important immunomodulatory role after allo-HCT, which may be at least partially dependent on the post-transplantation intestinal microbiome. Here we discuss the role of immune reconstitution in allo-HCT outcome, focusing on unconventional T cells, specifically mucosal-associated invariant T (MAIT) cells, γδ (gd) T cells, and invariant NK T (iNKT) cells. We provide an overview of the mechanistic preclinical and associative clinical studies that have been performed. We also discuss the emerging role of the intestinal microbiome with regard to hematopoietic function and overall immune reconstitution.

## Introduction

Allogeneic hematopoietic cell transplantation (allo-HCT) is performed as curative-intent therapy for numerous malignant and non-malignant hematologic diseases, as well as several immunological and metabolic disorders; however, its broader implementation is limited by high rates of transplantation-related complications and a 2-year mortality that approaches 50% (1). Key early contributors to this high post-treatment mortality are infection, the multi-system immunologic complication acute graft-versus-host disease (GVHD) and relapse of underlying malignancy. The most prevalent late contributors are chronic GVHD and organ dysfunction.

The primary goal of allo-HCT for hematologic malignancies is to harness the reconstituting donor immune system to recognize and eliminate residual tumor cells, therefore decreasing the probability of relapse, a phenomenon referred to as the graft-versus-leukemia effect (GVL) (2). Despite years of research in the field, meaningful separation of GVL effects from GVHD has been challenging, and it is thought that the same mechanisms underlie both forms of alloreactivity (3).

Acute GVHD arises when T cells in the donor graft recognize the recipient tissue as foreign. The pathology of acute GVHD is driven by direct cytotoxic effects of T cells as well as inflammatory cytokines, and commonly involves the skin, gastrointestinal tract and liver (4). Chronic GVHD is a late complication of allo-HCT and has different pathophysiology, characterized by chronic inflammation, dysregulated B cell and T cell immunity and later fibrosis (5). Research efforts in the field have improved outcomes for transplantation patients over the last several decades, but further work is required, particularly regarding post-transplantation immune recovery. Adequate reconstitution of the donor immune system—both innate and adaptive—is critical to patient outcome after allo-HCT for a number of reasons, namely, 1) early innate immunity is critical for tissue repair and infection control, 2) later restoration of adaptive immunity is key for responses to microbial and viral pathogens, 3) normal immune function is important for protective GVL effects, and 4) chronic GVHD is a syndrome best characterized by autoimmune-like dysregulation.

Successful immune reconstitution after allo-HCT depends on a number of factors, including the underlying malignancy, graft source, conditioning regimen, immune suppressive therapy for GVHD prophylaxis, GVHD itself when it occurs, and, of course, GVHD-directed therapies (6). Recipient age is another important factor, especially for *de novo* T cell generation due to age-associated thymic involution (7). In addition to these traditional modulators, evidence for the role of the gastrointestinal (GI) microbiome in shaping immune reconstitution following allo-HCT continues to emerge (8, 9) and is of growing interest specifically for microbiome-dependent unconventional T cell subsets, namely, the mucosal-associated invariant T (MAIT) cells, gamma delta (γδ) T cells, and invariant natural killer T (NKT) cells, all of which are thought to have a beneficial role in the post-transplantation setting. Therefore, in this review, we will discuss broadly the role of unconventional T cell subsets in allo-HCT and the potential relationship of the microbiota with hematopoietic function and peripheral immune reconstitution.

### Reconstitution of Innate Immunity

**Figure 1:**
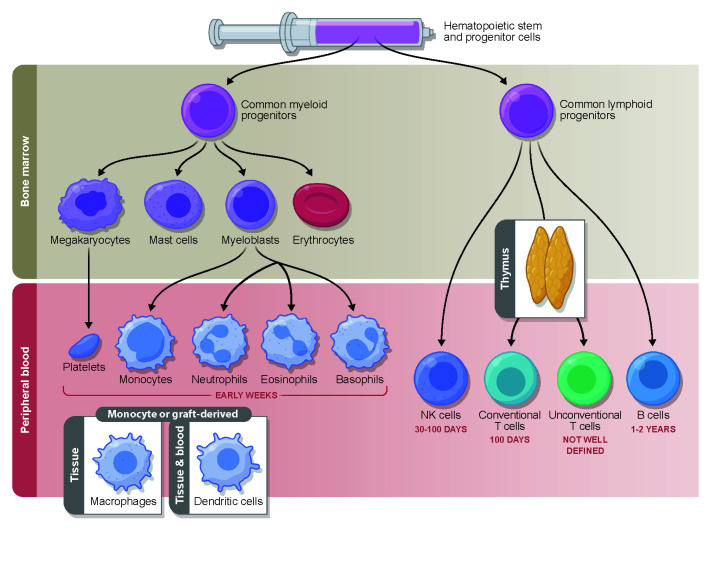
Timeline of immune reconstitution after allo-HCT. Myeloid reconstitution takes place in the early weeks post-transplantation, followed by the lymphoid compartment. NK cells typically return to steady state number first, followed by conventional T cells which reach pre-transplantation levels during the first 3 to 6 months. B cells commonly do not fully reconstitute until years after allo-HCT. The reconstitution of the unconventional compartment differs depending on the cell type and is a subject of ongoing investigation. The immune subsets measured in the periphery reflect cells from the donor graft that have been maintained and expanded (early) followed by true reconstitution of the hematopoietic compartment via the bone marrow progenitors transferred in the graft (which can occur quite early in the case of some myeloid lineages, but on a much longer time scale with respect to T cells that must undergo thymic education). While some post-transplantation reconstitutition mimicks immune system development in early life, there are many features unique to HCT.

Pre-transplantation conditioning and graft infusion are followed by a neutropenic phase. During this early phase after transplantation, the hematopoietic stem and progenitor cells infused with the graft differentiate and proliferate in the bone marrow to give rise to cells of both myeloid and lymphoid lineages (**Figure 1**). In the first 2 to 4 weeks after HCT, the descendants of myeloid progenitors, namely, neutrophils, eosinophils, basophils, and monocytes, appear in the peripheral blood and begin the reconstitution of the innate immunity. The first marker of innate immune recovery—neutrophil engraftment—is critical for anti-bacterial and anti-fungal immunity and the repair of conditioning-related tissue damage.

Natural killer (NK) cells represent the first, innate arm of the lymphoid lineage to reconstitute in the first weeks following allo-HCT (10) and comprise the majority of the peripheral blood mononuclear cells in this period. Due to their anti-tumor activity they are thought to be a crucial cell type in mediating GVL effects, which has been a subject of several recent review articles (11–14).

### Reconstitution of Adaptive Immunity and the Unconventional T Cell Populations

Adaptive immunity, required for appropriate responses to microbial and viral pathogens and vaccination is much slower to recover, and even when key cell types are present in normal numbers, their function is often impaired due to the endogenous alloreactive cytokine environment and exogenous immunosuppressive drugs, administered for the prevention or treatment of GVHD (6). T cells commonly reach normal counts in the peripheral blood in the first three to six months post-transplantation (CD8^+^ cells reconstitute faster than CD4^+^ cells), depending on the conditioning regimen and the choice of immune suppression (15). Two different processes contribute to the long-term T cell pool in post-transplant patients: initially, the T cells transplanted in the graft proliferate in the blood and peripheral organs of the lymphopenic recipient, and subsequently, lymphoid precursors from the transplanted stem cells are generated in the bone marrow and undergo selection in the recipient thymus. The latter truly *de novo* production of T cells begins after the recovery of the thymus from conditioning induced damage, and can be influenced by the further damage that occurs if GVHD develops (16). In contrast, B cells can remain at below-normal levels for years following transplantation. Several recent review articles have focused on the restoration of conventional immune subtypes after allo-HCT and their association with clinical outcomes (6, 7, 15, 17, 18), thus we will focus on other subsets here.

In recent years, more attention has been paid to the unconventional T cell subsets and their role in transplantation immunology and anti-tumor immunity. Unconventional T cells, namely, MAIT cells, γδ T cells, and iNKT cells, share features of both innate and adaptive immunity, specifically:

- Antigen-independent activation and rapid response similar to innate immune cells
- No donor MHC restriction, similar to innate immune cells
- TCR-dependent activation similar to conventional T cells, however, the TCR is semi-invariant and does not recognize conventional peptide antigens, but molecules presented in the context of monomorphic antigen presenting molecules

MAIT cells recognize bacterial metabolites of the riboflavin pathway presented by the class I like molecule MR1, and iNKT cells react to phospholipid antigens presented by another class I-like molecule, CD1d (19). Of note, in the setting of allo-HCT, this means that they are not restricted to either donor or host. γδ T cells represent a much more diverse population (from a TCR perspective) and various ligands have been described, which are specific to the combination of γ and δ chain and organ localization (19).

Our current understanding of the relationship of the microbiota to immune reconstitution after allo-HCT will be reviewed here, with a focus on what is currently known regarding the relationships of unconventional T cell populations with transplantation outcomes.

## The Intestinal Microbiome, Hematopoiesis, And Immune Reconstitution After Allogeneic Hematopoietic Cell Transplantation

The first indication that the commensal microbiota may influence hematopoiesis and migration of immune cells to sites of infection came from multiple studies performed in germ-free mice. Germ-free mice are known to have impaired hematopoiesis with fewer specific hematopoietic precursor cells of myeloid lineage, leading to impaired response to pathogens (20). Administration of the bacterial ligand NOD1 rescued impaired hematopoiesis in germ-free mice and induced production of hematopoietic cytokines in bone marrow mesenchymal stromal cells (21). Tada et al. observed lower neutrophil numbers in germ-free mice (22) and Inagaki et al. described impaired defenses against Listeria monocytogenes, which was attributed to defective trafficking of activated T cells to the sites of inflammation when compared to mice housed under specific pathogen free (SPF) conditions (23). Microbiota-driven myelopoiesis is dependent on functional toll-like receptor (TLR) signaling, as germ-free mice deficient in MyD88/TICAM signaling do not increase neutrophil generation upon microbial colonization (24). Similar effects have been observed with antibiotic treatment, which disrupts the normal microbiome. In mouse models of hematopoiesis and aging, oral broad-spectrum antibiotic treatment lead to impaired myelopoiesis and response to pathogens (20), depletion of circulating blood neutrophils (22, 25) and accelerated neutrophil aging (25). Josefdottir et al. demonstrated that the antibiotic-mediated microbiome changes led to decreased numbers and cell cycle activity in bone marrow progenitor cells and impaired maturation of granulocytes, and was dependent on functional Stat1 signaling in the bone marrow (26).

In addition to the potential influence on normal hematopoiesis, there is emerging preclinical evidence that the gut microbiota may influence clonal hematopoiesis (CH)—a recently defined condition, which represents a pre-leukemic state (27). A recent study by Meisel and colleagues utilizing a mouse model of TET2 deficiency (mimicking one of the commonly identified mutations in CH) suggested that mice with this genotype require impaired GI barrier function in order to develop CH. The key evidence for this was generated using 16S rRNA sequencing of peripheral blood, mesenteric lymph nodes and spleen, which confirmed higher bacterial burden in the organs of mice with TET2 deficiency than in age-matched controls. Furthermore, germ-free TET2 deficient animals failed to develop pre-malignant myelopoiesis and antibiotic administration reversed the CH phenotype. In symptom-free mice with TET2 deletion only in the hematopoietic cells (Tet2f/fVav^cre^ mice), but not in littermate controls, a pre-leukemic CH-like state could be triggered by administration of TLR2 agonist Pam3CSK4 (28). Further to this, hematopoietic stem cells express TLRs and appear to regulate emergency hematopoiesis in response to pathogen-derived signals (29, 30). Whether dysbiosis and CH are associated in humans remains to be explored.

In the clinical transplantation setting, using a large clinical data set of 1500 patients, 446 who had daily stool samples available, as well as daily complete blood counts, Schluter et al. demonstrated a relationship between peripheral blood lymphocyte, monocyte and neutrophil dynamics after allo-HCT and the intestinal microbiota composition. Patients who received fecal microbiota transplantation (FMT) in a randomized trial exhibited significantly higher white blood cell counts, which perhaps suggests a causal relationship between gut microbiome and circulating immune cell subsets (9). Ingham et al. observed faster B and NK cell reconstitution in patients with higher abundance of the bacterial family *Ruminococcacae*, which was associated with better clinical outcome (31). In a study from our group, Staffas et al. examined hematopoietic function in a mouse model of allo-HCT and demonstrated that the intestinal microbiota supports post-transplantation hematopoietic reconstitution in HCT recipients through its role in dietary energy uptake (8).

**Figure 2:**
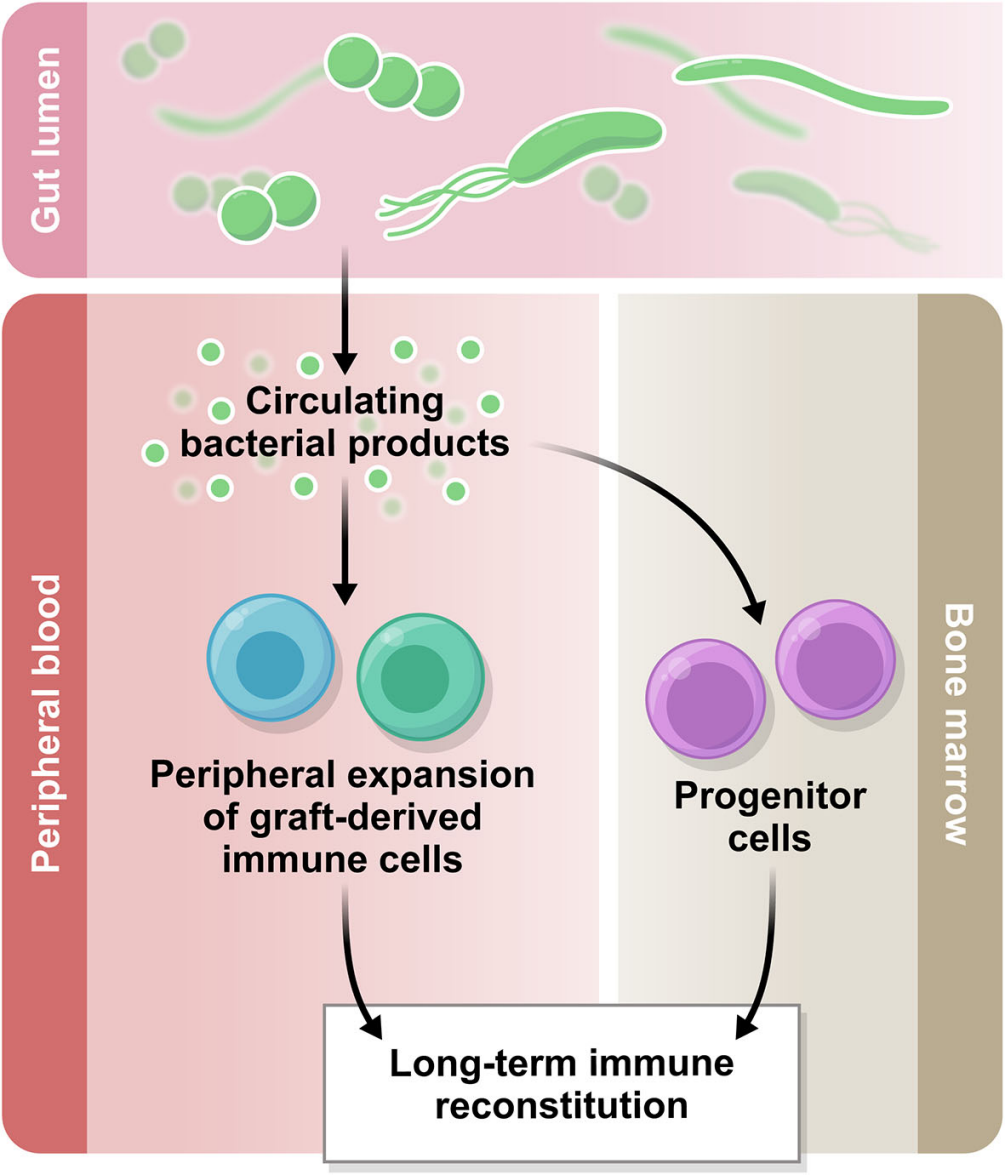
The gut microbiome in immune reconstitution. The intestinal microbiota produces a variety of metabolites which are biologically active. Some of these products circulate systemically and there is emerging evidence regarding their immunomodulatory potential. Based on the current literature, we hypothesize that the circulating bacterial products may influence peripheral immune reconstitution after allo-HCT.

In addition to the potential influence at the level of bone marrow resident progenitor cells, another possible mechanism for the microbiota to influence immune reconstitution and function may lie in circulating bacterial metabolites and other small molecules (**Figure 2**), for example short chain fatty acids (SCFA), bile acids, and aryl hydrocarbon receptor (AhR) ligands (32). There is currently no evidence that these molecules influence numeric reconstitution of any immune subset, but an emerging body of literature suggests they may influence immune function. We have recently reviewed the clinical associative data and detailed mechanistic studies supporting an immunomodulatory role for the microbiota in transplantation outcome in general (33). For example, butyrate, one of the short-chain fatty acids, has been associated with immunosuppressive effects and protection against GVHD in mouse models (34, 35), and in human post-transplantation samples, lower fecal concentrations of acetate, butyrate and propionate were associated with more severe acute GVHD (36). In a recent study from our group, butyrate and propionate concentrations in plasma were decreased at day 100 post-HCT in the patients who went on to develop chronic GVHD, supporting the hypothesis that these molecules are potentially immunomodulatory (37). Bile acids are another subset of microbiota-derived molecules with immunomodulatory potential. A recent study in mice has demonstrated a protective effect of tauroursodeoxycholic acid in acute GVHD due to a decrease in intestinal antigen presentation and the prevention of intestinal epithelial apoptosis (38). Furthermore, in a metabolomic analysis of plasma from allo-HCT patients, several bile acids, plasmalogens and aryl hydrocarbon receptor ligands appeared to be decreased in samples collected prior to acute GVHD development, compared with samples from patients who did not go on to develop acute GVHD (39). The molecules thought to influence conventional T cell fate (e.g., butyrate, which promotes T regulatory cells (Tregs) in mouse models of allo-HCT) have not yet been studied with respect to unconventional T cells in allo-HCT setting.

## MAIT Cells

### Introduction

MAIT cells are abundant in humans, preferentially localized in tissues and mucosa, and represent up to 10% of circulating CD3^+^ T cells in the peripheral blood and up to 45% of liver T cells (40). They are defined by their expression of a semi-invariant TCR α-chain (Vα7.2-Jα33/20/12 in humans, Vα19-Jα33 in mice), which can combine with only a limited number of TCR β-chains: Vβ2 (TRBV20) and V_β_13 (TRBV6) in humans and V_β_6 (TRBV19) and Vβ8 (TRBV13) in mice (41–44). MAIT cells respond to bacterial and fungal antigens presented in the context of the monomorphic MHC-class I-related molecule, MR1 (42).

Antigens presented by MR1 are thought to be predominantly derived from microbial vitamin B biosynthesis intermediates (45). These include activating vitamin B2 (riboflavin) metabolites, such as 5-(2-oxopropylideneamino)-6-d-ribitylaminouracil (5-OP-RU) (46), as well as non-activating 6-formylpterin (6-FP), a metabolite of vitamin B9 (folic acid) (45). An alternative route of MAIT cell activation is TCR-independent and occurs in response to IL-12 and IL-18 (47, 48).

### Development

MAIT cells develop in the thymus in a process that is strictly controlled by the gut microbiota. Bacterial ligand 5-OP-RU is produced by gut bacteria, circulates systemically, and is presented to MAIT cells in an MR1 dependent manner (49). This developmental process occurs during a narrow window in the early post-natal period and perturbations of this process (such as delayed bacterial colonization of the gut) lead to impaired MAIT cell development (50). The dependence of MAIT cell development on bacterial stimulation is also demonstrated by their low numbers in the thymus and the periphery of germ-free mice (42, 49, 51). During their development, MAIT cells differentiate into IFN-γ-producing T-bet^+^ MAIT-1 cells or the IL-17A-producing RORγt^+^ MAIT-17 cells (51, 52).

### Preclinical Data in Allogeneic Hematopoietic Cell Transplantation and Anti-Tumor Immunity

In the context of allo-HCT, a preclinical mouse model has demonstrated a role for recipient MAIT cells in preventing GVHD through production of IL-17A, promotion of intestinal barrier function, suppression of alloantigen presentation, and regulation of gut-microbiota composition (53). However, MAIT cells are a very rare population in mice, especially in circulation, where they represent less than 1% of CD3^+^ cells (53). The MAIT cell population can be clearly identified in mouse tissues but also in low frequency, making mouse studies of MAIT cells in allo-HCT very technically challenging.

Recently, several studies have examined the role of MAIT cells in anti-tumor immunity. Even though they displayed tumor-lytic capacity in *in vitro* assays (54–56), the most recent study demonstrated a tumor promoting function of MAIT cells. In this study MAIT cells promoted tumor growth and metastasis by blocking the effector function of NK cells and the therapeutic blockade of MR1 suppressed tumor growth and increased the immune cell infiltration in the tumor and their function (57).

### Clinical Data

In the clinical setting, several groups have now studied MAIT cell reconstitution after unmodified allo-HCT or cord blood transplantation. MAIT cell reconstitution after unmodified allo-HCT is poor and their numbers do not reach the levels seen in healthy controls, even after one to two years post-transplantation (58, 59) and their early presence in post-transplantation blood samples is at least in part dependent on the proliferation of MAIT cells transferred in the graft (58). Moreover, MAIT cells in the early post-transplantation period exhibit an altered phenotype in comparison to healthy controls with high CD69 and granzyme B expression and impaired IFN-γ and perforin response after bacterial stimulation *ex vivo* (59). Cord blood transplantation recipients exhibited even more delayed MAIT cell reconstitution (58, 60, 61) than recipients of peripheral blood stem cell (PBSC) grafts, with MAIT cell counts not reaching those of healthy controls for up to 5 years post-transplantation in children (60) and up to 10 years post-transplantation in adults (61).

Studies are emerging exploring the clinical association of MAIT cell reconstitution and acute GVHD; however, larger and more definitive studies are needed. Solders and colleagues observed a decrease in absolute MAIT cell counts in 22 patients with grade 2–4 GVHD after unmodified allo-HCT versus 16 patients with grade 0–1 GVHD, which correlated with the decreased absolute count in other lymphocyte subsets and was attributed to ongoing immunosuppression. The proportion of MAIT cells among CD3^+^ cells was unchanged (59). In a study of 17 pediatric cord blood transplantation recipients, subsequent MAIT cell reconstitution appeared unaffected by acute GVHD development (60). Bhattacharyya observed lower absolute MAIT cell counts on day 30 after allo-HCT in eight patients with grade 3–4 GVHD among total 105 patients. Moreover, presence of MAIT cells in a coculture assay suppressed CD4+ cell proliferation *in vitro* (58). In terms of MAIT cell reconstitution as a predictor of GVHD, lower absolute MAIT cell counts on day 60 (< 0.48 cells/ul blood) post-transplantation have been correlated with the development of acute GVHD in a multivariate analysis of 30 pediatric and adult patients receiving bone marrow (BM) transplantation (62). MAIT cell frequencies were lower in the patients with chronic GVHD following allo-HCT with unmodified grafts (63) and cord blood (61), though patient numbers were modest—22 and 98 respectively. Whether the relative loss of donor MAIT cells in acute and chronic GVHD is a biomarker for the GVHD process itself or has a functional role remains an open question.

Two studies thus far have examined the association of MAIT cell reconstitution with the post-transplantation microbiome composition. Bhattacharyya et al. observed a positive association between MAIT cell recovery and intestinal abundance of Blautia and Bifidobacterium in 54 patients with paired blood and stool samples at several timepoints after allo-HCT (58). In a separate study of 27 patients undergoing cord blood transplantation, Konuma et al. found higher microbial diversity and stool riboflavin pathway gene abundance in the early post-transplantation period in patients who exhibited MAIT cell reconstitution at six and twelve months compared to those with no reconstitution (61). Interestingly, the absolute numbers were still very low (approximately 0.1 cells/ul blood), similar to other published work regarding MAIT cells in cord blood transplantation (58, 60).

There are several factors implicated in affecting the post-transplantation reconstitution of MAIT cells. Definitive data regarding the influence of conditioning intensity on MAIT cell maintenance and development is not yet available, with conflicting results from several small studies that have been conducted thus far (58, 59, 62). With respect to the influence of GVHD prophylaxis, Bhattacharyya et al. observed markedly reduced MAIT cell number in patients receiving haploidentical transplantation with post-transplantation cyclophosphamide (PTCy), compared with expected MAIT count in the absence of PTCy (58). Furthermore, an analysis of MAIT cell reconstitution in association with cyclosporine A and glucocorticoid therapy in PBSC and cord blood transplantation recipients did not reveal any differences in MAIT cell number when patients who received these drugs were compared with those who did not (61).

Several studies have reported the presence of MAIT cells in solid tumor biopsy samples, including colorectal cancer (54, 55, 64, 65), kidney and brain cancer (66) and liver cancer (67). Results were heterogeneous, with increased intratumoral MAIT cells associated with both favorable and unfavorable clinical outcomes. Interestingly, a recent study identified a new MAIT cell subset in human colorectal carcinomas. These cells were directly stimulated by intratumoral bacterial antigens via their TCR and exhibited a distinct exhaustion phenotype, which was not observed in the MAIT cells from adjacent tissue or peripheral blood mononuclear cells (68). Regarding hematologic malignancies, MAIT cells have only been studied in multiple myeloma, where they exhibited lower counts in the blood (56, 69) and bone marrow (69) compared to healthy controls, and a higher expression of PD-1 (69). MAIT cell numbers were restored to baseline by PD-1 blockade (69). Of note, the process of mobilizing hematopoietic stem cells with G-CSF increased the numbers of IL-17-producing CD8^+^ MAIT cells in donor grafts, but association with persistence and patient outcome were not reported, therefore these remain interesting open questions (70).

Despite some contrasting results, early MAIT cell reconstitution seems to be associated with lower rates of acute GVHD development and better long-term MAIT cell reconstitution is associated with lower rates of chronic GVHD. Both early and late MAIT cell reconstitution appears to be dependent on intestinal microbiota as well as the predicted abundance of riboflavin genes (measured using Phylogenetic Investigation of Communities by Reconstruction of Unobserved States (PICRUSt)). Larger clinical studies are needed to clarify the association between peripheral MAIT cell reconstitution and acute GVHD. Similarly, more extensive stool data (including measured metabolites and MAIT cell-stimulating ligands) from allo-HCT recipients will be necessary to draw reliable associations between specific microbial taxa, genetic signatures and metabolite production and the reconstitution of MAIT cells after allo-HCT. The role of MAIT cells in the pro- versus anti-tumor immunity, which can be further applied to study their GVL activity is only beginning to emerge. 

## γδ T cells

### Introduction

Gamma delta T cells are another population of unconventional T cells, characterized by use of γ and δ chains instead of α and β within the TCR, as well as the use of only a limited number of V, D and J segments created during V(D)J recombination. Similar to MAIT cells, γδ T cell activation is not MHC- restricted (19). Of note, the usage of specific γ and δ chains is different in mice and humans. The Vδ chain usage is how the subsets of γδ T cells are defined (Vδ1, 2, 3, but in practical terms, Vδ2 vs. non-Vδ2 is how most analysis is divided, at least in studies of peripheral blood populations). In humans, γδ T cells comprise 0.5–10% of CD3^+^ T cells in the peripheral blood and the vast majority of these cells express the Vγ2Vδ9 receptor (19). These cells are activated by phosphoantigens, which are metabolites in the isoprenoid synthesis pathway. Isoprenoids are the oldest known biomolecules with numerous biochemical functions, including cell membrane synthesis, hormone synthesis and intracellular pathway regulation (71). This pathway is represented by the mevalonate pathway in mammalian cells, leading to the production of isopenthenyl pyrophosphate (IPP) (72) or the microbial 2-*C*-methyl-D-erythritol 4-phosphate (MEP) pathway with the intermediate 4-hydroxy-3-methyl-but-2-enyl pyrophosphate (HMBPP), which is the most potent activator of Vγ2Vδ9 cells (73). Phosphoantigens activate Vγ2Vδ9 cells by binding to butyrophilins (BTNs), particularly butyrophilin 3A1 (BTN3A1), which is ubiquitously expressed on almost all cell types and, unlike MHC class I, MHC class I–related molecule MR1 or antigen-presenting CD1 molecules, does not require presence of β2-microglobulin (74, 75). The so-called Vδ2-negative cells are predominantly located in tissues, such as skin, intestine, lungs, spleen, liver and uterus and form less than 10% of circulating γδ cells (76). In the peripheral blood, the non-Vδ2 population consists almost exclusively of Vδ1 cells, with Vδ3 cells accounting for only 0.2% of peripheral CD3^+^ cells (77). Vδ1 cells recognize various stress-related peptides and phospholipid antigens, including MHC-class-I-related ligands, such as stress ligands EPCR, MICA, MICB and ULBP and glycoproteins CD1c, CD1d (19).

### Development

In contrast to MAIT and iNKT cells, thymic γδ T cell development begins in the fetal period in both mice and humans. In mice, thymic egress occurs in waves, where each wave represents a different combination of γ and δ chains, has a different signature of surface and intracellular markers, and carries the predisposition to reside in different tissues. In humans, most of the cells developing in the fetal period are Vγ2Vδ9 cells, whereas the Vδ1 subset takes over in the postnatal period (78). 

### Preclinical Data in Allogeneic Hematopoietic Cell Transplantation and Anti-Tumor Immunity

The effector function of γδ T cells lies in their production of various cytokines and cytotoxic molecules. Their cytotoxic activity is mediated by production of perforin and granzyme, as well as expression of death receptor ligands, such as Fas-ligand and tumor necrosis factor-related apoptosis inducing ligand (TRAIL) (79). In mice, downstream cytokine production capacity is determined both during thymic development and by the environment (78). In humans, the majority of the thymic γδ T cells express IFNγ and TNFα but they can be polarized into IL-17 producing cells by exposure to IL-1β, IL-6, TGF-β, and IL-23 (80).

Following allo-HCT, γδ T cells mediate anti-viral, as well as anti-tumor effects and are thought to protect against relapse. Anti-tumor effects have been attributed to both Vδ1 and Vδ2 subsets via different ligands. Moreover, γδ T cells are capable of reactivity to multiple ligands on the tumor cells simultaneously using a single TCR and their early sensing of metabolic changes in the cells allows them to be among the first responders to malignant transformation (81).

Tumor cells can express an active mevalonate pathway of cholesterol synthesis and the metabolites of this pathway, such as IPP, may accumulate in the tumor cells and elicit a γδ T cell-mediated immune response (72). The enhancement of this process in the tumor cells by aminobisphoshonates and the subsequent expansion of tumor-reactive Vγ9Vδ2 cells has been demonstrated in several in vitro studies (82, 83), as well as in immunodeficient mice using human tumor cell lines and Vγ9Vδ2 cells (84). Besides the TCR, γδ T cells express several receptors also found on NK cells. Examples include NKG2D, CD94/NKG2C, DNAX accessory molecule-1 (DNAM-1), NKp30 and NKp44; and killer-inhibitory receptors (KIRs), like CD94/NKG2A, ILT2, CD161, or KIR2DL 1–3 (85).

NKG2D^+^ γδ T cells bind to tumors expressing NKG2D ligands, such as UL16-binding proteins (ULBPs) and MHC-class I related molecules MICA and MICB (86, 87). Activation of γδ T cells by NKG2D ligands can be initiated both via TCR and the NKG2D (88). Simultaneous activation of the TCR and DNAM-1 receptor of the Vγ9Vδ2 leads to killing of acute myeloid leukemia (AML) blasts *in vitro* and these cells enhance survival in a xenotransplantation murine model of leukemia (89). Leukemic stem cells, but not healthy CD34^+^ cells, redistribute BTN3A1 through the guanosine triphosphatase activity of RhoB, which enables their recognition by the Vγ9Vδ2 (90). Some hematological malignancies develop resistance against cytotoxic Vγ9Vδ2 via downregulation of ULBP1 (87). However, Vγ9Vδ2 are not the only subtype of γδ T cells capable of anti-tumor activity. Vδ1 cells can be cytokine-stimulated to express NKp30, NKp44 and NKp46, which are associated with cytotoxicity against lymphoid leukemia cells (91).

Another important mechanism for anti-tumor activity of γδ T cells is the expression Fc receptor FcRγIII (CD16). This receptor binds to the Fc portion of immunoglobulins and mediates anti-tumor effects via antibody-dependent cellular cytotoxicity (ADCC), similar to NK cells (92). In the case of γδ T cells, the ADCC is stimulated by phosphoantigen binding (93). Efficacy of the γδ T cell-mediated ADCC against CD19^+^ acute lymphoblastic leukemia was demonstrated using a CD19 antibody (94), as well as a so-called “triplebody” with 2 binding sites for CD19 and 1 for CD16 (95).

In experimental models of GVHD, multiple groups have demonstrated the alloreactive potential of γδ T cells, leading to GVHD development (96–98). Blazar and colleagues demonstrated that transgenic mice expressing gamma/delta heterodimers on a high proportion of peripheral T cells reacted to nonclassical major histocompatibility complex (MHC) class lb and caused acute GVHD when used as donors in allo-HCT mouse model (96). Maeda et al. observed reduced GVHD in mice treated with anti-γδ TCR antibody or in γδ deficient mice. This was explained by reduced donor T-cell expansion and reduced allogeneic stimulatory capacity of dendritic cells (DCs) (98). However, these mouse models have limitations for the study of γδ T cells due to differences in development, tissue distribution and other characteristics between mice and humans.

### Clinical Data

In contrast to αβ T cells, γδ T cells reconstitute early after allo-HCT (99–101). Whether the cells measured in patients following HCT with T cell replete grafts are generated *de novo* in the bone marrow and educated in thymus, or are a product of the *in vivo* expansion of γδ T cells transplanted in the graft has been a matter of debate. An older study from Hirokawa et al. observed common γδ TCR sequences between donor and host in selected patients in a study of 23 patients receiving allo-HCT (100). This finding has been supported by several studies linking graft γδ T cell content to their early reconstitution and clinical outcomes after allo-HCT (102–106). However, a more recent study by Ravens et al. shows both similar and different γδ TCR clonotypes in the donor-recipient pairs, suggesting that the post-transplantation γδ T cell reconstitution may occur both de novo in the bone marrow and thymus, as well as via peripheral graft expansion (99). Vδ2 cell reconstitution was significantly impaired in the recipients of cord blood transplantation, whereas Vδ1 counts appeared to be driven by CMV reactivation and did not differ between cord blood and unmodified stem cell graft recipients (107).

The role of γδ T cells after allo-HCT can be viewed from several perspectives. First, γδ T cells exert potent anti-infectious immunity against a multitude of bacteria and viruses. In a cohort of 102 pediatric patients, higher γδ T cell counts post-transplantation were associated with lower incidence of bacterial, fungal, and viral infections (108). Serial monitoring of Vδ2 counts post-transplantation has found an association between high Vδ2 counts and lower rates of EBV reactivation (109). Patients with higher numbers of γδ T cells in the early post-transplantation period (day 30) experienced less CMV reactivation than patients with lower γδ T cell numbers (104). Similarly, patients with higher CD27+ γδ T cell counts in the graft had lower rates of CMV reactivation than those with lower numbers (103). A further study from the same group also demonstrated a role of CD8^+^ γδ T cells, which were higher in the grafts from CMV positive donors, expressed V*γ*9 and exhibited increased reactivation to cytokine and TCR/CD3 stimulation (105). In addition to these associations with less CMV reactivation, γδ T cells expand in response to CMV, which suggests an involvement in viral clearance (99, 106, 110–112). Ravens et al. demonstrated that this expansion is clonal and the clones proliferating in the context of CMV reactivation carry virus-reactive γδ TCR sequences (99). Anti-CMV activity has mostly been attributed to Vδ2-negative subsets (predominantly Vδ1) of γδ T cells (99, 110, 111). Vδ1-positive γδ T cells were also demonstrated to undergo clonal expansion in the context of EBV reactivation (113, 114).

The anti-tumor activity of γδ T cells predicts a protective role after allo-HCT with respect to relapse. The first study published in line with this hypothesis showed that among 43 patients undergoing T-cell depleted HCT from partially HLA-mismatched donors for leukemia, 10 achieved a ‘high’ γδ T cell proportion—as defined by reaching a proportion greater than 10% of total CD3^+^ cells on two consecutive measurements post-transplantation. This correlated with improved disease-free survival (DFS) up to 30 months post-transplantation, where 90% of patients with increased γδ T cell proportion were disease free, compared to 31% of patients with normal proportion of γδ T cells (115). In a follow-up study, Lamb et al. confirmed the findings after 42 months of follow-up in an additional cohort of 100 patients, comparing transplantation outcomes using two different *ex vivo* α/β T cell depletion regimens. Moreover, they demonstrated that Vδ1 cells are the major subset in the patients with robust γδ T cell reconstitution and moreover, these cells exhibit anti-leukemia activity *in vitro* (116). In a follow up report, with an extension of the follow-up period to 8 years and an expansion of the cohort, the 5-year overall and disease-free survival was significantly improved in the group with higher γδ T cells (117). In the most recent study of 108 patients undergoing BM or PBSC transplantation, Minculescu et al. linked improved γδ T cell reconstitution on day 56 post-transplantation to a significantly decreased cumulative incidence of relapse and improved overall and relapse-free survival. When the counts of all γδ T cells and their subsets individually were analyzed as continuous variables, increased numbers of all γδ T cells subsets correlated with decreased risk of death and increased numbers of all γδ T cells and Vδ2 cell subset correlated with lower risk of relapse (118). The CMV-expanded clones of non-Vδ2 cells isolated from patients after unmodified allo-HCT, as well as cord blood transplantation, showed efficacy in killing leukemic blasts in vitro (111). Consistent with this finding, Dolstra et al. had previously demonstrated that Vδ1 cells isolated from a patient after allogeneic HCT exhibited an anti-tumor activity against AML blasts. Similar to NK cells, the leukemia-reactive γδ T cells expressed killer cell-inhibitory receptor (KIR) p58.2 (CD158b) (119). Not all γδ T cells harbor the same GVL efficacy. Gaballa et al. attributed this effect to CD8^+^ γδ T cells in the graft (103), and Jin et al. to oligoclonal expansion of the *TRDV4* and *TRDV8* subfamilies in patients after allo-HCT. In contrast, *TRDV5* and *TRDV6* clones were higher in patients experiencing recurrence of the disease (120). Further to this, Arruda studied the TCR repertoire of γδ T cells in the donor graft and identified that patients without relapse more commonly received a graft containing γδ T cells with a higher proportion of ‘public’ TCRs in the repertoire. However, in contrast to Scheper et al. (111), γδ T cells in grafts derived from CMV positive donors displayed a more private, less diverse, skewed repertoire (121).

Early clinical studies linked higher γδ T cell counts to a higher incidence of acute GVHD, whether measured in the recipient (122) or in the graft (123), but also reported decreased γδ T cell counts in patients with chronic GVHD, specifically the CD4 and CD8 double negative subset (124). The association of γδ T cells with acute GVHD was not confirmed in further human studies, which associated γδ T cell reconstitution only with enhanced GVL effect and not higher GVHD incidence (108, 115–117). Higher γδ T cell counts on day 28 post-transplantation have been associated with lower risk of acute GVHD, when both the whole γδ T cell population is measured, or just the Vδ2 cell subset (118). Additionally, lower counts of naïve γδ T cells in the donor grafts have been associated with subsequent development of grade 2–4 acute GVHD in the recipient (125). It has been postulated that this protective effect may be due to to a regulatory subset of FoxP3 expressing γδ T cells (126, 127). Interestingly, specific subsets and clones have been proposed to be differentially responsible for GVHD and GVL effects. Gaballa et al. recently associated higher GVHD incidence with one specific subset of γδ T cells, which were CD8^+^. In a cohort of 105 patients, those receiving grafts with higher CD8^+^ γδ T cell numbers experienced higher incidence of grade 2–4 acute GVHD, but in parallel, a perhaps predictable lower incidence of relapse (105). Additionally, in a 2005 study of 13 patients receiving allo-HCT for multiple myeloma TCR spectratyping led to the observation of unique γδ T cell clones associated with GVHD and new dominant TCR peaks associated with clearance of the IgH clones, supportive of some tumor-specific γδ T cell responses, but not definitive (128).

Several immune profiling studies of patients transplanted with grafts depleted of αβ T cells have demonstrated an association between early γδ T cell reconstitution and positive transplantation outcome. Evidence for the minimal contribution of γδ T cells to GVHD comes from clinical success of performing transplantations with αβ T cell depleted grafts (129), as well as using these grafts as a ‘stem cell boosting strategy’ in the setting of graft failure (130). Airoldi and colleagues observed rapid Vδ1 and Vδ2 T cell reconstitution in 27 pediatric patients receiving haploidentical αβ^+^ T and CD19^+^ B cell-depleted grafts. Vδ1 cells expanded *in vivo* in the context of CMV reactivation, whereas Vδ2 cells exhibited activity against leukemia blasts *in vitro* (101).

Extensive literature describes the beneficial role of γδ T cells in the post-transplantation period but factors influencing their reconstitution, outside of viral reactivation, have not been described in a detailed fashion. Of note, their reconstitution does not appear to be dependent on conditioning intensity (118), however, γδ T cells appear extremely sensitive to PTCy in the setting of haploidentical transplantation. In this setting, γδ, and especially the Vδ2^+^ T‐cell counts were significantly lower in the early post-transplantation period (131, 132). This effect on the Vδ2^+^ cell population persisted for up to one year post-transplantation and correlated with more frequent EBV reactivation (131).

An interaction between the intestinal microbiota and γδ T cells has been proposed in mouse models of several diseases, largely for the intraepithelial populations of γδ T cells (as opposed to the more easily measured circulating cells). Intraepithelial γδ T lymphocytes are reduced in germ-free mice and can be induced after colonization of these mice (133). In a mouse model of lung adenocarcinoma, intestinal microbiota induced intrapulmonary IL-17-producing Vδ1 cells, which promoted inflammation and tumor progression (134). In an additional mouse model of ischemic stroke, the intestinal microbiota appeared to modulate central nervous system inflammation via IL-17 producing γδ T cells (135). To our knowledge, no associations have yet been drawn between the intestinal microbiota and circulating γδ T cells. Given the previous preclinical data, as well as the reactivity of the Vδ2 subset to bacterial metabolite HMB-PP, the gut microbiome may play a role in γδ T cell reconstitution following allo-HCT.

## iNKT Cells

### Introduction

The term natural killer T (NKT) cells was originally assigned to a group of CD3^+^ cells expressing markers found on the NK cells, such as CD161. However, further research demonstrated that these markers do not fully define this population, which responds to lipid molecules presented by the MHC-I-like molecule CD1d (19, 136). There are two broad subtypes of NKT cells. Type I NKT cells, also called invariant NK T (iNKT) cells, are characterized by an invariant TCRα chain (typically Vα14-Jα18 in mice and Vα24-Jα18 in humans), accompanied by a limited number of TCRβ chains (mainly Vβ8.2, Vβ7 and Vβ2) (136–138). Type I NKT cells recognize α-galactosylceramide presented by CD1d molecule, can be recognized by α-GalCer-loaded tetramers and are the most studied subtype to date. Type II NKT cells also react to lipid molecules presented in the context of CD1d, but they are not reactive to α-GalCer and bear more diverse TCRs than type I NKT cells (138). iNKT cells are also more abundant in mice [up to 50% of the liver and bone marrow T cells (139)] than in humans [representing only about 0.1% of peripheral blood T cells (19)]. iNKT cells recognize either self-lipids or foreign lipids produced by pathogenic or commensal bacteria, fungi, viruses or present in allergens. Upon activation, they rapidly gain effector function with cytotoxic activity, with transcription factor and cytokine production dependent on tissue localization and acquire either Th1, Th2 or Th17 phenotype (140, 141). Similar to MAIT cells, aside from TCR-dependent activation, iNKT cells can be activated by cytokines, such as IL-12 (140).

### Development

iNKT cells develop postnatally in the thymus where they encounter the CD1d molecule expressed by double positive cortical thymocytes, in a process that requires intracellular trafficking of lipid antigens presented by CD1d (78). In contrast to MAIT cells, the commensal microbiota are not vital for thymic iNKT cell development (142).

### Preclinical Data in Allogeneic Hematopoietic Cell Transplantation and Anti-Tumor Immunity

In mouse models of allo-HCT, recipient iNKT cells ameliorate GVHD. Early studies examining the role of iNKT cells in mouse models of allo-HCT assessed for the effect of reduced intensity conditioning (RIC) together with total lymphocyte irradiation (TLI) and anti-thymocyte globulin (ATG) on GVHD development. Mice receiving this treatment regimen experienced less GVHD, as well as an expansion of iNKT cells, which was not observed in the CD1d deficient mice. The protection from GVHD was mediated through increased Th2 polarization of donor T cells (143). Later studies further explored the mechanism by which the iNKT cells reduce GVHD. They studied GVHD development in CD1d and Jα-18 deficient mice, which represent more specific models to assess for the invariant portion of NKT cells. iNKT cells reduced the expansion of alloreactive donor T cells in the GVHD target organs (144), as well as promoted the expansion of the protective Treg population in an IL-4 dependent manner (145). Protection from GVHD has also been observed upon adoptive transfer of iNKT cells in mice, both of host and donor origin, as well as third-party (146–151). Effects of adoptive transfer of human CD4^+^ and CD4^-^ iNKT cells into NSG mice have also been examined in a xenogeneic GVHD model. CD4^-^ iNKT cells inhibited GVHD by decreased human T cell activation and Th1 and Th17 polarization. CD4^+^ and CD4^-^ iNKT cells induced dendritic cell (DC) maturation, but CD4^-^ iNKT cell contact with splenic and monocyte-derived DCs was more intense and associated with more iNKT cell degranulation (152). In a chronic GVHD model, adoptive transfer of iNKT cells demonstrated a protective role for this cell type, and even reversed the chronic GVHD phenotype (153). Similarly, several groups demonstrated protective effect of α-GalCer administration in acute and chronic GVHD models (146, 153–156).

Extensive studies have been performed examining the role of iNKT cells in tumor immunology. The first evidence of their anti-tumor activity comes from the study of Crowe et al. in the setting of methylcholanthrene induced sarcoma, where mice lacking iNKT cells were more susceptible to tumor development (157). Since then, numerous studies have explored the role of iNKT cells in the immune surveillance of various tumors, mostly attributing them an anti-tumor activity (158). The evidence is somewhat thinner in the context of hematologic malignancies, however, CD1d has been shown to be expressed on multiple myeloma cells (159, 160), as well as AML cells (161) and iNKT exhibited reactivity to CD1d positive tumor cells *in vitro* in a α-GalCer–dependent manner. In line with these findings, iNKT cells from donor lymphocyte infusion (DLI) could be expanded *ex vivo* and were capable of lysing leukemia cell lines and patient AML cells in CD1d-dependent manner (162).

### Clinical Data

Several studies have addressed iNKT cell reconstitution post-transplantation in human subjects, largely focusing on associations with GVHD. In the first published study of a cohort of 106 patients, Haraguchi et al. observed iNKT cell reconstitution within a month after allo-HCT in PBSC graft recipients, but their numbers remained very low in the first post-transplantation year in the bone marrow (BM) graft recipients. Peripheral blood iNKT cell counts were lower in patients experiencing acute and chronic GVHD (163). In another study, iNKT cell reconstitution was examined at multiple timepoints after allo-HCT using a CD1d tetramer in a cohort of 71 patients cohort who received a mixture of reduced intensity and myeloablative conditioning regimens (RIC and MAC), either BM or PBSC grafts, and who had a variable exposure to *in vivo* T cell depletion with ATG. In both univariate and multivariate analysis, reaching a threshold of iNKT/T cell ratio higher than 10^-3^ in at least one of multiple measurements on day 15, 30, 60 and 90, was an independent predictor of lower incidence of acute GVHD and better overall survival (164). A more recent study from the same center focused on different cell populations in 117 BM and PBSC grafts (HSCs, NK cells, conventional and regulatory T cells and iNKT cells) and observed that the iNKT cells were the only population associated with lower incidence of grade 2–4 acute GVHD in a univariate analysis. In the multivariate analysis, only the lower frequency of CD4^-^ iNKT cells could predict higher incidence of acute GVHD in patients receiving BM and PBSC grafts and higher CD4^-^ iNKT cell *ex vivo* expansion capacity was associated with lower rates of grade 2–4 GVHD in patients receiving PBSC grafts (165). In line with these findings, Bosch et al. observed a positive correlation between graft iNKT cell numbers and peripheral iNKT cell reconstitution of the host (166). Chaidos et al. showed that higher than median CD4- iNKT cell graft content is protective against grade 2–4 GVHD. Moreover, CD4- iNKT cells were capable of contact inhibition of T cell proliferation and suppressed their IFNγ secretion *in vitro* (167). This indicates that iNKT cell reconstitution post-transplantation might be dependent on the expansion of the graft-derived population rather than de novo production in the bone marrow. Several groups have studied the association of conditioning and anti-thymocyte globulin (ATG) administration with immune reconstitution and, in addition to examining other cell subsets, also characterized iNKT cell reconstitution. Total lymphocyte irradiation (TLI) and ATG administration following RIC appeared to favor iNKT cell maintenance or development, and patients conditioned in this fashion had lower incidence of acute GVHD (168). Interestingly, these findings did not hold true in the setting of MAC, where Servais et al. did not observe any difference in iNKT cell numbers when comparing patients receiving ATG versus no ATG (169). Bosch et al. even observed an extremely slow iNKT cell reconstitution after ATG administration after MAC and a significant correlation between graft iNKT cell numbers and peripheral iNKT cell reconstitution of the host on both early and late timepoints post-transplantation (166). These results suggest extremely slow endogenous recovery of iNKT cells after MAC. PTCy in the setting of haploidentical transplantation appears to be another factor associated with the rate of iNKT cell reconstitution, with lower counts at day 30 and day 90 compared in patients receiving PTCy compared with other graft types (132). The association of early iNKT cell reconstitution and clinical outcome might be correlated with the presence of other immune subtypes. Kim et al. correlated lower frequencies of iNKT cells and monocytic myeloid derived suppressor cells measured before day 30 with higher incidence of grade 3–4 GVHD in a multivariate analysis of 119 recipients of unmodified (111 patients) and cord blood (eight patients) grafts (170).

Based on these data, a clinical trial was performed testing a liposomal formulation of α-GalCer (RGI-2001) in recipients of allo-HCT. Patients receiving this ligand on the day of transplantation exhibited improved reconstitution of Helios^+^ Treg cells (defined as higher than 12% of CD4^+^ cells at any timepoint after allo-HCT) and lower incidence of grade 2–4 acute GVHD (171).

Both peripheral and intratumoral iNKT cell counts have been investigated in several different malignancies (e.g., head and neck squamous cell carcinoma, lung cancer, colorectal cancer) and higher numbers have largely been associated with better prognoses (172). With regard to hematologic malignancies, iNKT cells have been most studied in multiple myeloma. Their counts inversely correlated with disease progression (159, 173–175), which was linked to decreasing CD1d expression on the tumor cells as the disease progressed (160). In a small study of 6 patients, α-GalCer-pulsed dendritic cells expanded the iNKT cell population when added to lenalidomide therapy and led to a decrease of the monoclonal immunoglobulin in asymptomatic myeloma patients (176). In AML, low iNKT cell counts were correlated with poor overall survival (177). Despite the evidence of anti-tumor activity of iNKT cells in the context of hematologic malignancies, no difference in relapse incidence after allo-HCT has been observed in association with iNKT cell counts (164, 165).

Although commensal microbiota are not vital for thymic iNKT cell development, iNKT cells are an important intermediary in the relationship between host and microbiota, especially on the mucosal surfaces and in the liver. Examples include identification of distinct bacteria and less intestinal inflammation in the in the *J*α*18^-/-^*mice versus**wildtype mice in the experimental model of colitis (178). In the human colonic biopsies from patients with inflammatory bowel disease, iNKT cells produced pro-inflammatory cytokines, which was driven by exposure to mucosa-associated microbiota (179). Exposition to pathogenic bacteria or gentamicin led to decrease in hepatic NKT cells and higher degree of liver injury (180). In metastatic colon cancer, changes in the gut microbiota potentiated iNKT-cell mediated tumor control via decreased secondary bile acid production (181). To date, the influence of gut microbiota on circulating NKT cells and their reconstitution after allo-HCT has not been clarified.

Based on the literature reviewed here, iNKT cells represent a cell subset associated with protection against acute GVHD in allo-HCT patients. iNKT cells might harbor a GVL potential, which was not demonstrated in the published studies, possibly due to their extremely low numbers post-transplantation. Administration of an iNKT cell ligand proved beneficial in a small number of patients. Therefore, further exploration of iNKT cell expansion *in vivo* are needed to harness their anti-GVHD and anti-tumor potential. Their reactivity to changes in commensal microbiota in other disease models makes them a good candidate for treatments exploring microbiota modifications, however, more research is needed in this area.

## Discussion

Unconventional T cells represent populations are emerging as likely important for the field of transplantation immunology with a potential to reduce the risk of acute and chronic GVHD without impairing, or perhaps improving in the case of γδ T cells, GVL effects. Thus far, published studies linking unconventional T cell subtypes to favorable clinical outcomes are limited by low patient numbers, and variations in type of malignancy, conditioning regimens, graft types and immune suppressive drugs. Therefore, further studies are needed. Manipulation of unconventional T cell compartment may improve clinical outcomes in the future - for example, selecting grafts with high unconventional T cell numbers, exogenous administration of specific ligands, using adoptive transfer approaches or microbiota manipulation strategies. Some of these approaches are currently being investigated in clinical trials, others still require more mechanistic studies to gain deeper understanding. Moreover, the interplay of different types of unconventional cells with each other and their conventional counterparts might also play a role in reconstitution. For example, the Vδ2 subset shares a number of characteristics with MAIT cells, i.e. they can both undergo cytokine-dependent activation and share similar transcription profiles, thus they have some shared post-transplantation requirements for function and maintenance (182).

As described above, for each unconventional cell subtype there is evidence for their function being modulated by intestinal microbiota. However, it is unclear whether the bacteria-derived ligands of unconventional T cells circulate in the blood or execute their functions only in the context of cell contact with antigen presenting cells in the tissues. However, there has been emerging evidence in recent years demonstrating that circulating bacterial metabolites can be associated with disese outcomes. Although more evidence is needed, it is possible that these metabolites influence circulating immune cells, both numerically and functionally. Similarly, the number of studies linking the intestinal microbiome to hematopoiesis is increasing. Given that bone marrow is a remote organ to the gut, circulating bacterial metabolites might be one explanation for this phenomenon. Nevertheless, circulating immune cells likely reflect, in some fashion, the cell distribution within the organs, specifically the unconventional T cells, which are unique regarding their immediate reactivity to bacterial metabolites. MAIT cell reconstitution post-transplantation has been linked to higher abundance of certain bacteria in the gut, as well as the presence of vitamin B2 metabolic pathways in the gut bacteria in a small number of patients. For γδ T cells and iNKT cells, the evidence of their dependence on gut microbiota or organ specific microbial flora is only beginning to emerge in mouse models of diseases unrelated to allo-HCT. Further studies are needed to clarify the dependence of the reconstitution of not only of MAIT cells, but of all unconventional subsets on the presence of distinct microbial taxa and metabolites. We hypothesize that one of the reasons that microbiota damage is associated with poor overall transplantation outcome (183) is due to the influence of microbial communities on the reconstitution of robust immunity, a hypothesis we hope will be studied in detail.

## Author Contributions

HA reviewed the literature, designed the figures and wrote the manuscript. KM contributed to the figure design and wrote the manuscript. MB critically revised the manuscript. All authors contributed to the article and approved the submitted version.

## Funding

This research was supported by NCI awards, MSKCC Cancer Center Core Grants P30 CA008748, R01-CA228358 (MB), R01-CA228308 (MB), P01-CA023766 (MB); NHLBI award R01-HL125571 (MB), R01-HL123340 (MB); NIA National Institute of Aging award Project 2 of P01-AG052359 (MB); NIAID award U01 AI124275 (MB); Tri-Institutional Stem Cell Initiative award 2016-013 (MB); The Lymphoma Foundation (MB); The Susan and Peter Solomon Divisional Genomics Program (MB); and the Parker Institute for Cancer Immunotherapy at Memorial Sloan Kettering Cancer Center (KM, MB); KM wishes to acknowledge funding received from DKMS and the Parker Institute for Cancer Immunotherapy. HA wishes to acknowledge funding received from the Deutsche Forschungsgemeinschaft (DFG).

## Conflict of Interest

The authors declare that the research was conducted in the absence of any commercial or financial relationships that could be construed as a potential conflict of interest.
